# High resting energy expenditure in women with episodic migraine: exploring the use of predictive formulas

**DOI:** 10.3389/fnut.2023.1296937

**Published:** 2023-11-24

**Authors:** Laís Bhering Martins, Jéssica Sales Ribeiro, Ana Maria dos Santos Rodrigues, Luana Caroline dos Santos, Antonio Lúcio Teixeira, Adaliene Versiani Matos Ferreira

**Affiliations:** ^1^Department of Nutrition, Universidade Federal de Minas Gerais, Belo Horizonte, Brazil; ^2^Department of Psychiatry and Behavioral Sciences, The University of Texas Health Science Center, Houston, TX, United States

**Keywords:** resting energy expenditure, migraine, predictive formulas, pain, nutrition

## Abstract

**Introduction:**

Migraine is a common and disabling primary headache, and its pathophysiology is not fully understood. Previous studies have suggested that pain can increase humans’ Resting Energy Expenditure (REE). However, no previous study has investigated whether the REE of individuals with migraine differs from the general population. Therefore, this study aims to assess whether the REE of women with migraine differs from that of women without headaches. We also tested the accuracy of REE predictive formulas in the migraine patients.

**Methods:**

This cross-sectional study involves 131 adult women aged between 18 and 65 years, 83 with migraine and 48 without (controls). We collected clinical, demographic, and anthropometric data. Migraine severity was measured using the Migraine Disability Test and Headache Impact Test, version 6. The REE was measured by indirect calorimetry, and it was compared with the predicted REE calculated by formulas.

**Results:**

Patients with migraine had higher REE when compared to controls (*p* < 0.01). There was a positive correlation between REE and the patient-reported number of migraine attacks per month (Rho = 0.226; *p* = 0.044). Mifflin-St Jeor and Henry and Rees were the predictive formulas that have more accuracy in predicting REE in women with migraine.

**Discussion:**

Considering the benefits of nutritional interventions on treating migraines, accurately measuring REE can positively impact migraine patient care. This study enhances our understanding of the relationship between pain and energy expenditure. Our results also provide valuable insights for healthcare professionals in selecting the most effective predictive formula to calculate energy expenditure in patients with migraine.

## Introduction

Migraine is a common and disabling primary headache. Migraine attacks last from four to 72 h and are typically characterized by unilateral location pulsating quality, moderate or severe intensity, associated with nausea, photophobia, and phonophobia, and can be accompanied by visual disturbance called aura (1).

The migraine pathophysiology is not fully understood. One widely accepted theory suggests that endogenous or exogenous factors can promote the activation of trigeminal afferent fibers that innervate cranial structures. Trigeminal activation induces the release of vasoactive neuropeptides by sensory fibers triggering a cascade events with the release of inflammatory mediators such as bradykinin and prostaglandins, leading to neurogenic inflammation (2).

Previous studies have shown that pain can increase the Resting Energy Expenditure (REE) in humans (3–6). For instance, Holland-Fischer et al. (5) evaluated the effect of non-traumatic skin pain caused by transcutaneous painful electrical stimulation on REE and energy substrate utilization in 10 healthy volunteers. Pain caused an acute and reversible increase by 62% in REE, probably mediated by adrenergic activity and increased muscle tone. Older studies involving critically ill patients have also shown that pain increases energy expenditure, while analgesia and sedation decrease energy expenditure (3, 4, 6). Recent studies have further suggested that pain increases energy expenditure during routine daily activities (7, 8). In a study conducted by Ko et al. (7), older adults with knee pain consumed more oxygen while walking than those without pain, indicating an increase in energy expenditure. However, the association between energy expenditure and pain remains a largely unexplored area in the literature.

Despite the evidence suggesting the effect of pain on REE, no previous study has investigated whether the energy expenditure of individuals with migraine differs from the general population. However, a clear understanding of the pattern of energy expenditure in patients with migraine is relevant for the adequacy of dietary interventions, especially knowing that nutritional intervention may provide benefits in treating migraines (9–12).

Therefore, the primary objective of this study was to assess whether the REE of women with migraine differs from that of women without migraine. The secondary aim was to investigate the accuracy of predictive formulas to determine REE in women with migraine.

## Methods

This cross-sectional study involves adult women aged between 18 and 65 years, both with a diagnosis of migraine and without (control). We chose to include only women in our sample due to the higher prevalence and greater severity of migraine among this population (1, 13).

A neurologist diagnosed patients with migraine according to the International Classification of Headache Disorders - 2nd edition (1). Exclusion criteria were: (i) Other Headaches (not characterized as migraine); (ii) Any chronic health conditions (e.g., diabetes mellitus, arterial hypertension, chronic renal failure, and endocrine diseases such as hypo- or hyperthyroidism); (iii) Pregnancy and breastfeeding; (iv) Smoking, and (v) Alcohol abuse (> 2 doses/day). Patients were evaluated during a headache-free period.

This study took place at the Headache Outpatient Clinic of Hospital das Clínicas, Universidade Federal de Minas Gerais (UFMG) (Belo Horizonte, Minas Gerais, Brazil). Ethical approval for this study was obtained from the Human Research Ethics Committee of UFMG (CAAE: 28236814.3.0000.5149), and all procedures were conducted in accordance with the principles of the Declaration of Helsinki.

### Demographic and clinical data

Clinical and demographic data (age, sex, marital status, years of education, physical activity in minutes per week, and the patient-reported number of migraine attacks per month) were collected using a semi-structured questionnaire specifically created for this study. Migraine severity was measured using the Migraine Disability Test (MIDAS) and Headache Impact Test, version 6 (HIT-6). The MIDAS provides information about the impact of headaches on the number of days lost in social, domestic, and work activities in the last 3 months. The MIDAS scores were classified into: no or little disability (a score up to 5), mild disability (a score from 6 to 10), moderate disability (a score from 11 to 20), and severe disability (a score above 21) (14). The HIT-6 measures the incapacity caused by headaches in the previous month. The HIT-6 scores were classified into: little or no impact (scores up to 49), some impact (a score from 50 to 55), substantial impact (a score from 56 to 59), and very severe impact (a score above 60) (15).

### Anthropometric evaluation

The body weight and height were measured, and the Body Mass Index (BMI = weight/height^2^) was calculated. Patients were classified as follows: Underweight (BMI ≤ 18.5), Normal Weight (BMI from 18.5 to 24.9), Overweight (BMI from 25.0 to 29.9), or Obesity (BMI ≥ 30) (16).

After an overnight fast, body composition was evaluated by electric bioimpedance using a Biodynamics^®^ (model 310e) device. The test was performed in a comfortable and quiet room. The patients were instructed to: (i) not to exercise at least 24 h before and to abstain from alcoholic beverages, coffee, or black tea (ii) to remain to lie still throughout the test. The percentage of fat mass was classified according to the categories proposed by Shea et al. (17).

### Assessment of resting energy expenditure

As validated by previous studies, REE was defined by Indirect Calorimetry using the MetaCheckTM device (model 7,100; Korr Medical Technologies, Salt Lake City, UT, United States). Patients underwent the evaluation in the morning after overnight fasting. Patients were seated in a comfortable position and breathing through a disposable mouthpiece or mask attached to the device. Nasal clips were used to ensure that all air passed through the mouthpiece. Patients were instructed to relax, breathe normally, avoid coughing, and minimize their movements until the test was completed. The test duration was approximately 20 min. Oxygen consumption was measured, and the REE was calculated using the equation proposed by Weir with an assumed respiratory quotient of 0.83 (18).

Since body composition affects REE (19), the measured REE (mREE) was corrected for lean body mass (mREE/FFM). The correction was made by dividing the mREE (in kcal/dia) by fat-free mass (in kilograms).

Finally, mREE was compared with the predicted REE (pREE) calculated using the formulas presented in Table 1. These formulas were selected based on their research and clinical relevance (20–30).

**Table 1:** Predictive equations used to estimate Resting Energy Expenditure in women with migraine.

References	Predictive equations
Harris and Benedict	655.1 + 9.56 x W + 1.85 x H + 4.67 x A
Mifflin-St Jeor	9.99 x W + 6.25 x H− 4.92 x A −161
WHO/FAO/UNU W	18–30 years = 14.7x W + 496 31–60 years = 8.7 x W + 829 >60 years = 10.5x W + 596
WHO/FAO/UNU WH	18–30 years = 13.3 x W + 334x H* +35 31–60 years = 8.7 x W − 25x H* +865 >60 years = 9.2 x W + 637x H* −302
Owen	795+ 7.18 x W
Schofield	18–30 years = (0.062 x W + 2.036) x 239 30–60 years = (0.034 x W + 3.538) x 239 60 years = (0.038 x W + 2.755) x 239
Cunningham	(22 x FFM) + 500
Henry and Rees	18–30 years = (0.048 x W + 2.562) x 239 30–60 years = (0.048 x W + 2.448) x 239

A, age in years; H, height in centimeter (cm) or H*, height in meters (m); W, weight in kilograms; WHO/FAO/UNU W considering just weight in kilograms; WHO/FAO/UNU WH considering height and weight; FFM, fat-free mass in kilograms.

### Statistical analyses

Categorical variables were described as frequencies (%) and compared using Chi-square or Fisher’s exact test. The distribution of quantitative variables was tested using the Kolmogorov–Smirnov for analyses of all the groups (sample size > 50) and Shapiro–Wilk tests for analyses just with the data of the migraine group (sample size < 50) (31). Variables with a normal distribution (parametric) are presented as means (±standard deviation) and non-parametric variables are presented as medians (minimum-maximum). Independent Sample *t*-test and Mann–Whitney were used to compare of mean and median between groups, respectively. Pearson’s or Spearman’s coefficients were calculated to assess the correlation between parametric and non-parametric variables, respectively. A Binary Logistic Regression test was performed to adjust the comparison of mREE/FFM values between groups by weekly minutes of physical activity. Paired Student’s *t*-test (parametric variables) and Wilcoxon’s test (non-parametric variables) were used to compare mREE and pREE.

In addition, the 95% confidence interval (95% CI) of the difference between pREE and mREE was calculated. The pREE was considered accurate when it was between 90 and 110% of the mREE at the individual level. pREE values lower than 90% or higher than 110% of mREE were classified as underestimated and overestimated, respectively (20, 32–35). The mean percentage difference between pREE and mREE (bias) was considered the measure of accuracy at the group level and the individual accuracy was determined ≥50%. The level of agreement between mREE and pREE was examined using frequencies and quartile similarities and the Bland and Altman method (36). We use the Linear Regression analyses for investigated the proportionality bias between the formulas.

The statistical analyses were performed using SPSS, version 19 (IBM Corp., Armonk, NY, United States) and *p* < 0.05 was considered statistically significant.

Our simple size was enough to obtain a statistical power greater than >90% for mREE/FFM, considering the simple size minimum of 48 individuals per group, independent sample *t*-test, and alpha error of 5%.

## Results

### Clinical data

Eighty-three women diagnosed with episodic migraine and 48 controls were enrolled in the study. The presence of aura was identified in 59.0% (*n* = 49) of the patients with migraine. The median (range) age was 30 (19–65) and 33 (18–65) years old for the migraine and control groups, respectively (*p* = 0.101). Most participants were single and had graduate degrees (*p* = 0.477 and *p* = 0.445 comparison between groups, respectively). The groups were similar in body composition parameters (Table 2). Most patients with migraine were classified with “moderate to severe disability” according to MIDAS (67%) and “substantial or very severe impact” according to HIT (94%).

**Table 2:** Body composition of women with and without migraine.

Characteristics	Control group (*n* = 48)	Migraine group (*n* = 83)	*p*-value
Body fat mass (%)	32.1 (±5.7)	31.5 (±5.6)	0.572^a^
**Classification of body fat mass**			
Low weight	0 (0.0)	1 (1.2)	0.856^b^
Normal	28 (58.3)	49 (59.0)	
Overweight	17 (35.4)	26 (31.3)	
Obesity	3 (6.3)	6 (7.2)	
**Classification of body mass index**			
Eutrophy	22 (45.8)	50 (60.3)	0.279^b^
Overweight	19 (39.6)	24 (28.9)	
Obesity	7 (14.6)	9 (10.8)	
Activity physical (min/week)	170.0 (0–900)	47.5 (0–840)	0.008^c^

^#^Parametric numerical variables are represented as mean (±standard deviation) and non-parametric variables as median (minimum - maximum).

a Student *t*-test.

b Chi-squared test.

c Mann-Whitney.

**Figure 1:**
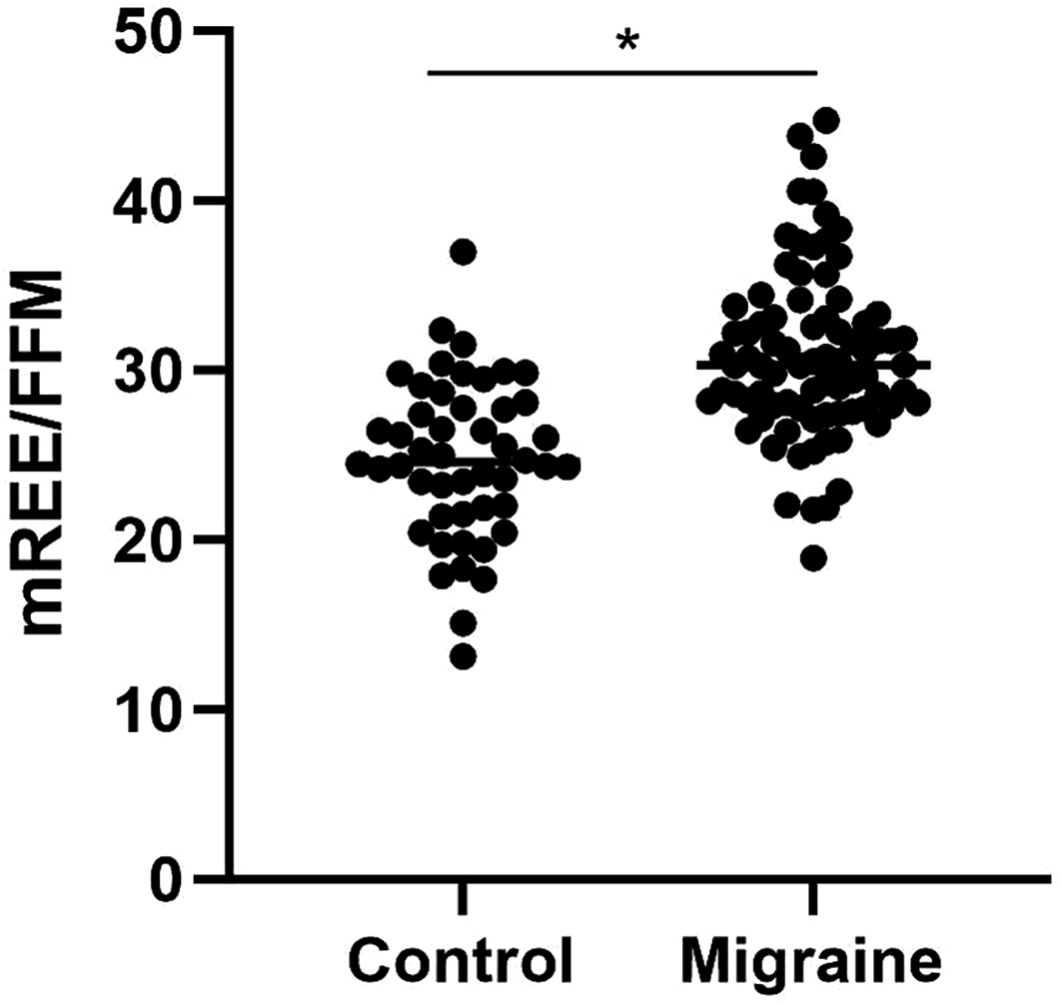
Comparison of resting energy expenditure per kilogram of fat-free mass (REE/FFM) between women with and without migraine. The symbol * in the figure represents that there was a statistical difference (*p*<0.001).

Patients with migraine had higher mREE/FFM when compared to controls (30.92 ± 4.99 vs. 24.51 ± 4.34, respectively; *p* < 0.001) (Figure 1). The mREE/FFM positively correlated with the number of migraine attacks per month (Rho = 0.226; *p* = 0.044), but not with HIT (Rho = 0.080, *p* = 0.472) and MIDAS (Rho = 0.114, *p* = 0.313). The physical activity was different between groups, however, the difference in mREE/FFM between the groups remains after adjustment for minutes of physical activity per week (*p* < 0.001).

### Predictive formulas to estimate REE

Considering that the mREE of women with migraine differed from women without migraine, we evaluated the accuracy of REE predictive formulas for this population.

The mean mREE of female patients with migraine was 1326.22 (±243.75) kcal/day. Only 25% of the formulas were able to predict the REE similar to the mREE. The Mifflin-St Jeor formula had the lowest mean difference (7.69 ± 184.52) kcal/day compared to mREE. Individual accuracy (precision) was >50% for the Mifflin-St Jeor, Owen, Schofield, and Henry and Rees formulas.

The formula that most underestimated the REE was Owen’s (30.1%), while Cunningham (54.2%), Harris & Benedict (47.0%), and FAO WH (45.8%) overestimated the REE as shown in Table 3.

**Table 3:** Comparison between resting energy expenditure measured by indirect calorimetry and calculated by predictive formulas in women with migraine.

		**Difference**			**Prediction**			
	**kcal/dia**	***p*-value**	**kcal/dia**	**95% IC**	Accuracy % (n)*	**Underestimation % (n)**	**Overestimation % (*n*)**	**† (%)**
Calorimetry	1326.22 (243.75)	–	–	–	–	–	–	–
Harris and Benedict	1425.36 (126.74)	<0.001	−99.14 (183.06)	−139.11; −59.17	44.6 (37)	8.4 (7)	47.0 (39)	−7.48
Mifflin-St. Jeor	1333.90 (147.47)	0.705	−7.69 (184.52)	−47.98; 32.60	60.2 (50)	18.1 (15)	21.7 (18)	−0.58
WHO/FAO/ONU W	1413.86 (155.45)	<0.001	−87.64 (183.25)	−127.65; −47.63	48.2 (40)	7.2 (6)	44.6 (37)	−6.61
WHO/FAO/ONU WH	1409.84 (151.68)	<0.001	−83.62 (184.60)	−123.93; −43.31	44.6 (37)	9.6 (8)	45.8 (38)	−6.31
Owen	1256.32 (89.84)	0.002	69.88 (197.64)	26.73; 113.04	56.6 (47)	30.1 (25)	13.3 (11)	5.27
Schofield	1402.98 (155.83)	<0.001	−76.76 (184.23)	−116.99; −36.53	51.8 (43)	7.2 (6)	41.0 (34)	−5.79
Cunningham	1444.34 (144.85)	<0.001	−118.80 (203.27)	−163.47; −74.14	34.9 (29)	10.8 (9)	54.2 (45)	−8.91
Henry and Rees	1337.26 (143.68)	0.585	−11.05 (183.44)	−51.10; 29.00	57.8 (48)	15.7 (13)	26.5 (22)	−0.83

^#^Numeric variables are presented as mean (standard deviation).

^##^Statistical differences were assessed using the Wilcoxon test.

^*^Accuracy was considered when pREE fell between 90 and 110% of mREE (individual level).

^†^(%) represents the percentage mean of the difference between pREE and mREE (group level).

**Figure 2:**
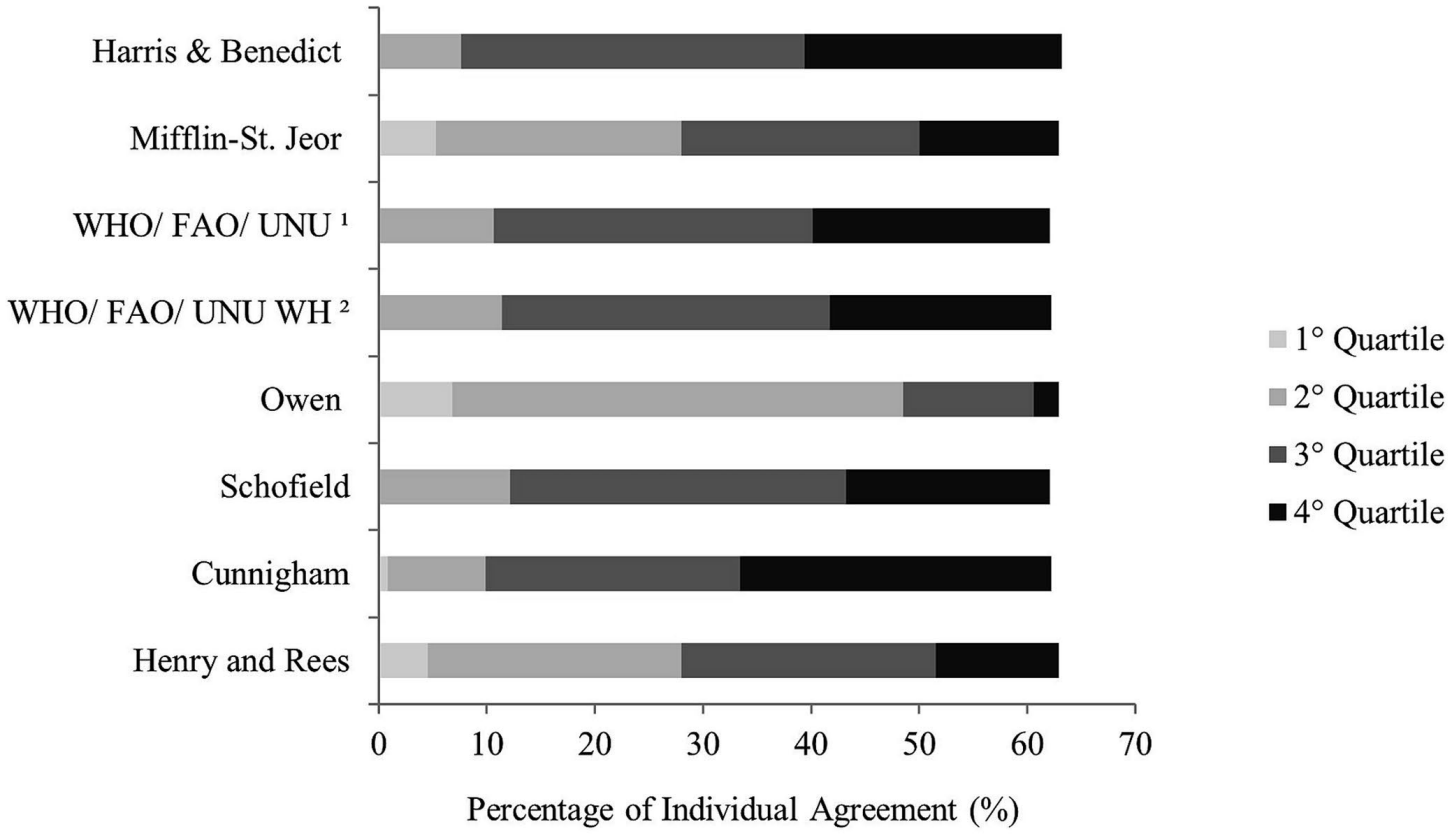
Percentage of individual agreement between resting energy expenditure measured by indirect calorimetry and predictive formulas by quartile in women with migraine.

Figure 2 shows the individual agreement between mREE and pREE by quartile similarity in women with migraine. All formulas presented individual accuracy higher than 60%. Four equations (50.0%) showed no agreement between the mREE lowest quartile. Cunningham showed the highest individual agreement for the highest values measured by IC (28.80%). The pREE by Mifflin-St. Jeor and Henry and Rees and Owen showed individual agreement for all quartiles of the mREE.

## Discussion

In this study, we showed that women with migraine had a higher REE when compared to individuals without migraine. The mREE/FFM positively correlated with the number of migraine attacks, indicating that the more episodes of pain, the higher the patient’s REE. Regarding the predictive formulas, Mifflin-St Jeor and Henry and Rees were those that have more accuracy to predict REE in patients with migraine. To the best of our knowledge, this is the first study to evaluate differences in the REE in patients with migraine and the adequacy of predictive formulas.

Our findings are in line with the aforementioned previous studies that suggested that pain increases energy expenditure (3–6). In our study, we observed a positive correlation between mREE/FFM and the frequency of migraine attacks. In our study, we observed a positive correlation between mREE/FFM and the frequency of migraine attacks. This indicates that higher energy expenditure is linked to the frequency of pain rather than its severity, as assessed by HIT and MIDAS, in patients with episodic migraines. Indeed, previous studies have suggested the presence of an energy metabolism disturbance in migraines, potentially caused by mitochondrial dysfunction, which may be associated with increased neuronal excitability and susceptibility to migraines (37–39). Interestingly, a recent study observed a high prevalence of lifetime migraine (61%) among patients affected by mitochondrial disorders and suggested that migraine may be a clinical manifestation of brain energy dysfunction (37). Our findings corroborate the theory that migraine is associated with energy metabolism disturbance. However, more studies are needed to explore the link between mitochondrial dysfunction, REE, and migraine. Furthermore, it’s worth noting that in our present study, patients were evaluated during pain-free periods. Therefore, another intriguing aspect to explore in future investigations is the relationship between REE and pain intensity during migraine attacks.

Nutrition plays a crucial role in migraine pathophysiology, as certain foods have been identified as triggers for migraine attacks (40). Furthermore, overweight has been linked to the severity of migraines and is influenced by diet (12). Accurately predicting REE can assist dietitians and nutritionists in providing more effective guidance on healthier dietary patterns for patients with migraines. While indirect calorimetry is considered the gold standard method for determining REE, its use in clinical practice is limited due to cost, time requirements, and the need for trained staff to perform the examination. Therefore, predictive equations remain the most used tool for estimating REE (20, 41, 42). In this study, we assessed the accuracy of various predictive formulas in determining REE in patients with migraine. Our findings suggest that the Mifflin-St Jeor and Henry and Rees formulas demonstrated the highest accuracy at the individual level, lower mean percentage differences at the group level, and better representation in classifying adequacy by quartile for the migraine group. Cunningham’s (28) formula was included to explore whether using lean mass in the calculation improves formula accuracy. However, our results indicated that Cunningham’s formula had the highest percentage of overestimation of mREE in women with migraine. Although Cunningham’s formula was originally developed using data from participants of the Harris-Benedict study (i.e., predominantly normal-weight women, excluding male athletes), previous studies have shown high accuracy when this formula is applied to estimate REE in athletes (43, 44). Considering that migraine patients, in general, may have less muscle mass than athletes, the coefficient of 22 in the Cunningham formula may be excessive for them, leading to an overestimation of REE.

The distinct characteristics of the reference population used to establish the predictive formulas may be a reason that justifies the discrepancy between them. REE can be influenced by several demographic, clinical, and environmental factors, including sex, age, race, physical activity level, diet, body composition, obesity-related comorbidities, and environmental temperatures (19, 45). Of note, the reference population used to develop Henry and Rees formula was composed by mostly normal-weight individuals with varied ethnicities and from tropical countries, resembling our sample. On the other hand, the Mifflin-St Jeor equation was developed based on a sample that included individuals of both sexes, ranging from 19 to 78 years old, with normal weight and overweight, from a US cohort. The accuracy of these formulas in comparison to indirect calorimetry to measure REE was tested in various groups within the Brazilian population, yielding mixed results. While most studies suggested that the Henry and Rees or Mifflin-St Jeor formulas tend to overestimate REE in a Brazilian sample of adults (46, 47), patients with severe obesity (48, 49), nonalcoholic fatty liver disease (50), postmenopausal women (51), and adolescent football athletes (52), results from other studies involving patients with pulmonary hypertension (53), type 2 diabetes (54), and liver transplant recipients (55) indicated an underestimation of these formulas when compared with REE measured by calorimetry.

Of note, in the current study, we used a reduced steady-state system to assess REE. We opted for this approach since previous research had employed the same equipment to assess the reliability of predictive formulas within the Brazilian population (35, 56). Nevertheless, other systems that measure a five-minute steady state over a 30-min interval may offer a more precise REE measurement (57). Given that this is the initial study to evaluate the accuracy of predictive formulas in individuals with migraine, further investigations employing different REE measurement systems are warranted to validate our findings.

The current study is subject to certain limitations, including its cross-sectional design, the exclusive inclusion of women with episodic migraine in the sample, and the absence of biomarkers such as triiodothyronine and thyroxine that are associated with REE. However, to ensure the validity of our findings, we carefully selected participants by excluding individuals with metabolic diseases and thyroid abnormalities. Furthermore, as aforementioned, we chose to include only women in our sample due to the higher prevalence and greater severity of migraine among women (1, 13). Therefore, to further explore the relationship between REE and pain mechanisms in patients with migraine, future longitudinal studies encompassing both men and women are warranted.

Given the potential impact of nutritional interventions in reducing the impact and severity of migraines (12), it is crucial to have a reliable tool for determining resting energy expenditure (REE) when developing personalized dietary intervention. In clinical practice, the selection of the most accurate REE predictive formulas specific to a particular population is essential. Our findings not only support existing literature on the relationship between pain and increased energy expenditure but also demonstrate that the Mifflin-St Jeor and Henry and Rees predictive formulas are particularly accurate for women with migraines. However, further longitudinal studies involving chronic migraine patients and other pain-related disorders are needed to validate these results.

## Data availability statement

The raw data supporting the conclusions of this article will be made available by the authors, without undue reservation.

## Ethics statement

The studies involving humans were approved by Human Research Ethics Committee of UFMG (CAAE: 28236814.3.0000.5149). The studies were conducted in accordance with the local legislation and institutional requirements. The participants provided their written informed consent to participate in this study.

## Author contributions

LM: Conceptualization, Data curation, Investigation, Methodology, Supervision, Writing – review & editing, Funding acquisition. JR: Conceptualization, Data curation, Writing – original draft, Funding acquisition. AR: Data curation, Writing – original draft. LS: Data curation, Writing – original draft. AT: Writing – review & editing. AF: Writing – review & editing, Conceptualization.
